# The Effects of Animal, Collection Time, and Interval on the Microbiota Structure, Metabolism, and Degradative Potential of Rumen Fluid Inoculum Collected by Esophageal Probe from Hay-Fed Cows

**DOI:** 10.3390/ani14233547

**Published:** 2024-12-09

**Authors:** Marica Simoni, Alexandros Mavrommatis, Andrea Cresceri, Marco Severgnini, Mauro Penasa, Matteo Santinello, Bianca Castiglioni, Paola Cremonesi, Eleni Tsiplakou, Federico Righi

**Affiliations:** 1 Department of Veterinary Science, University of Parma, Via del Taglio 10, 43126 Parma, Italy; marica.simoni@unipr.it (M.S.);; 2 Department of Animal Science, Agricultural University of Athens, Iera Odos 75, GR-11855 Athens, Greece; 3 Institute of Biomedical Technologies, National Research Council (ITB-CNR), 20054 Milan, Italy; 4 National Biodiversity Future Center, 90133 Palermo, Italy; 5 Department of Agronomy, Food, Natural Resources, Animals and Environment, University of Padova, Viale dell’Università 16, 35020 Padova, Italy; 6 Institute of Agricultural Biology and Biotechnology, National Research Council (IBBA-CNR), 26900 Lodi, Italy

**Keywords:** in vitro digestibility, rumen microbiota, enzymes, fiber analysis

## Abstract

Rumen fluid (RF) is the greatest source of uncontrolled inter-assay variations in in vitro digestibility tests. This study aimed to investigate the effect of donor cow (DC) individual variability, time relative to feeding, and weekly sampling intervals on RF microbiota structure and metabolism, and on its degradative capacity expressed as enzymatic activity (EA). RF was collected using an esophageal probe from 4 Holstein hay-fed DCs before feeding, and 4 and 8 h post-feeding, over 3 days with one-week intervals, for a total of 3 weeks. Rumen bacteriome ß-diversity was affected by DCs and the one-week interval but not by time relative to feeding. However, volatile fatty acids and NDF and DM digestibility were not altered based on DCs. Propionic, lactic, and butyric acids were consistent between weeks, with some variations in lactic and butyric acids 8 h after feeding. Amylase and cellulase activities fluctuated between the weekly intervals, while xylanase activity increased in RF collected at 4 and 8 h after feeding (*p* = 0.030) and in DC3 compared to the other DCs (*p* < 0.001). Our results reinforce the importance of pooling RF from multiple cows and increasing the number of replicates to enhance the accuracy of the analysis.

## Simple Summary

In vitro digestibility tests are essential for the evaluation of the nutritional value of forages and feeds but they need rumen fluid collected from animals as inoculum to be performed. However, it is well known that the degradative capacity of the rumen fluid can fluctuate depending on the animal and the time of collection, probably as a function of the rumen microbes’ populations. The enzymatic activity expressed by the microbes is expected to represent the rumen fluid’s degradative potential. The objectives of the present study were to investigate the effects of the animal and sampling time and interval on rumen microbes’ populations and degradative potential but also to investigate the relationship between different rumen characteristics. Rumen microbes differed by animal and week but not daytime. Rumen function indicators were not affected by the animal and only minor microbes’ metabolites showed variations during the day. The enzymatic activity was more variable depending on the animal and time, indicating the need to mix rumen fluids and replicate the analysis to obtain trustable results.

## 1. Introduction

Various in vitro laboratory techniques have been developed to estimate the dry matter digestibility (DMD) and neutral detergent fiber digestibility (NDFD) of forages and fiber-rich feeds, with the aim to apply precise feeding strategies in ruminants and/or to select plant cultivations with higher nutritional value [1]. In these in vitro techniques, the enzymatic degradation of plant fibers is achieved using an inoculum, which represents a major source of variability in the analysis results. Rumen fluid (RF), fecal, and artificial enzymatic inocula have been discussed as starters in a recent review on the outcomes of digestibility methods and trials [2]. Amongst the above, RF is the more commonly used inoculum. However, several collection methods may be adopted, all with different strengths and weaknesses. The oral stomach tubing method is a less invasive alternative compared to cannulation, which is the reference method. Among the weaknesses of this collection technique, there is a high probability of saliva contamination [3] and the disproportionate exclusion of solid particles containing adherent bacteria [4]. There is a paucity of studies that have accomplished optimizing enzymatic activity (EA) or incubation conditions concerning artificial enzymatic inocula, while fecal inoculum has been demonstrated to express a lower fibrolytic potential compared to RF [5].

Feeding donor animals with standardized diets, collecting inoculum at a fixed time relative to feeding, and pooling RF from multiple donor animals are the main strategies to control the variability of the rumen inoculum activity and consequently its fermentative consistency [6]. Historically, the use of cannulated donor cows (DCs) that were fed forages was only recommended when the NDFD must be tested in fibrous feeds [7]. However, cattle cannulation is not always allowed due to animal welfare issues [8], so the use of esophageal probes becomes the main option to collect RF from DCs, despite its potential disadvantages such as possible saliva contamination, which affects microbial and enzyme dilution.

Although RF has been adequately assessed as an inoculum as mentioned above, it also represents the greatest source of variation in in vitro digestion trials. The EA and the microbial structure of the inoculum have shown significant variations in different ruminant species, breeds, productive stages, individuals, and within the same animal at different time intervals, with the donors’ diet and feeding pattern being the main factors affecting the variability and the repeatability of the outcomes, along with RF handling [9,10,11]. In a recent study, even though more than 80% of the core rumen microbiota of dairy cattle were found to be identical, the VFA production and the EA of xylanase, protease, and amylase were significantly affected by the dietary CP, NDF, starch levels, and the animals’ age (1.5 to 27 mo old) [12].

Another crucial factor that affects both the rumen microbiota structure and its EA is the time relative to feeding. More specifically, the EA of CM-cellulase, xylanase, and amylase showed an upward trend with their peaks 3 h post-feeding in the rumen of cows fed a TMR containing corn as the main source of starch and alfalfa hay and wheat straw as the main sources of NDF [13]. Moreover, it has been reported that monthly sampling intervals result in different rumen microbiota structures due to seasonal changes, with the main variables driving these changes being the temperature and humidity [14,15]. An in-depth understanding of the factors affecting rumen inocula microbiology, biochemistry, and EA, along with their interconnections relative to the individual animal, time after feeding, and period of collection, is of interest to standardize, validate, and possibly certify their use for analytical purposes.

The objective of the present study was to investigate the effects of the animal, sampling time, and interval on the microbiota structure and degradative potential of RF inoculum collected by esophageal probes from hay-fed cows. A further objective was to investigate the relationship between the parameters used to characterize RF to provide insights into their capacity to express the inocula’s degradative potential.

## 2. Materials and Methods

### 2.1. Experimental Design

Rumen fluid inocula were collected by specialized veterinary personnel using esophageal probes from 4 randomly selected Italian Holstein DCs (2 primiparous: DC1 and DC2; and 2 multiparous: DC3 and DC4) 3 times per day for 3 days with one-week intervals. The pH of RF was measured by a portable pH meter Checker (HANNA instruments Italia Srl, Padova, Italy). At the same intervals, feces were collected from each cow after perineal stimulation. The cows were permanently dry and fed once a day with grass hay only, which was then available ad libitum in the manger. Botanical species were in prevalence, including *Avena fatua*, *Dactylis glomerata*, *Festuca arundinacea*, *Phleum pratense*, and *Lolium multiflorum.* The trial was performed in the middle of the fall season and the temperature humidity index detected during the 3 consecutive weeks was on average 61.9, 55.6, and 58.6. On each day, RF were collected before the main meal, which was administered at 8:00 a.m. (T0), after 4 h (T4), and after 8 h (T8) (Table 1). After filtration through 4 layers of cheesecloth, performed under carbon dioxide flow, the liquid fraction of each sample was divided into 3 aliquots: the first was freeze-dried and stored at −80 °C for the microbiota analysis; the second was frozen at −20 °C for VFA determination; and the third was prepared for the EA determination. The latter involved centrifugation of samples at 5000× *g* for 15 min, filtration of the supernatant through 0.45 µm porosity PVDF syringes filter, collection in plastic tubes, and storage at −20 °C for the EA determination. The solid fraction that remained on the cheesecloths was stored at −80 °C for the microbiota analysis as well.

### 2.2. NDF and DM Total Tract Digestibility Estimation

Hay and fecal samples were oven-dried at 55 °C for 48 h (pre-drying) and then ground in a hammer mill (Retsch S/S Cross Beater Hammer Mill Sk1, Haan, Germany) to pass a 1 mm screen. The chemical composition of the hay was determined as follows: an aliquot of 5 g of pre-dried sample was oven-dried at 103 °C overnight to measure the DM content. Ash content was determined by ignition at 550 °C for 4 h. The CP content was analyzed by the Kjeldahl method, and the ether extract was determined by the Soxhlet–Henkel method [16]. Fiber fractions were analyzed sequentially. The amylase-treated neutral detergent fiber (aNDF) was assayed with heat-stable amylase without sodium sulfite and expressed inclusive of residual ash, and ADF was expressed inclusive of residual ash [17]. Hemicellulose content was calculated as difference between aNDF and ADF, whereas cellulose content was calculated as difference between ADF and ADL. The non-fiber carbohydrates (NFCs) were calculated as 100 − (NDF + CP + ether extract + ash) and expressed as % DM. In vitro aNDF digestibility at 24 h of fermentation and the undigestible NDF after 240 h of fermentation (uNDF) of hay and fecal samples were determined through the in vitro fermentation system [7,18]. The total tract apparent dry matter digestibility (ttaDMDe) and the estimated total tract neutral detergent fiber digestibility (ttNDFDe) were calculated for each cow at each time and interval using the average uNDF analysis of the hay fed to the animals and those of feces [19]. For the fermentation process, RF was collected from the same 4 cows, pooled, and processed as described in the literature [20]. Briefly, the collected RF was kept at 39.5 °C under anaerobic conditions and was blended and filtered through 4 layers of cheesecloth. Rumen fluid was inoculated at the ratio 1:4 to the medium in a flask containing 0.5 g of sample and incubated for 24 and 240 h. The chemical composition of the hay is reported in Table 1.

### 2.3. DNA Extraction, Purification, and Next-Generation Sequencing

Bacterial DNA was extracted from rumen liquid and solid fraction samples following the method proposed by [21] using the repeated bead beating plus column method. An amount of 0.250 g of lyophilized sample was added in a 2 mL glass tube containing zirconium beads (0.3 g of 0.1 mm and 0.1 g of 0.5 mm) and added with 1 mL of lysis buffer (500 mM of NaCl, 50 mM of Tris-HCl, 50 mM EDTA, 4% sodium dodecyl sulfate, pH 8). Cell lysis was obtained by mixing the glass tubes at high speed for 3 min using the TissueLyser II system (Qiagen, Hilden, Germany). After cell lysis, 10 M ammonium acetate was used to precipitate and remove impurities while, after centrifugation at 16,000× *g* for 10 min at 4 °C, the supernatant was recovered in 2 aliquots of 1.5 mL Eppendorf along with isopropanol (Sigma-Aldrich, Saint Louis, MO, 33539-1L-R) to recover nucleic acids. RNA and proteins were removed using proteinase K and buffer AL, followed by the use of QIAamp columns provided by Qiagen DNA Stool Mini Kit (Qiagen, Hilden, Germany). The quali-quantitative analysis of the extracted DNA was performed using spectrophotometer (Nanodrop ND-1000, Thermo Fisher Scientific, Waltham, MA, USA) at 260 and 230 nm of wavelength. The specific primers used to PCR amplify the V3-V4 regions of the 16S rRNA gene were FOR: CCTACGGGNGGCWGCAG and REV: GACTACHVGGGTATCTAATCC. The primers were selected following the literature [22]. Purification was performed by AmPure XP bead (Agencourt, Beverly, MA, USA). Two primers were used from the Nextera XT Index kit and amplification was performed using 25 µL of KAPA HiFi HotStart ReadyMix (Kapa Biosystems, Inc., Wilmington, MA, USA). Library quantification was performed employing Agilent Bioanalyzer 2700 (Agilent DNA 7500, GE Healthcare, Piscataway, NJ, USA). The concentration of library pool was determined by fluorimeter (Qubit 3 Fluorimeter, Invitrogen, Thermo Fisher Scientific, Waltham, MA USA) and libraries were then sequenced by Illumina MiSeq instrument with a paired-end 2 × 300 run.

### 2.4. Bioinformatics Analysis

Raw sequencing reads were processed, generating a single fragment covering the whole amplicon from the two overlapping pairs, using PandaSeq software (v2.5, Masella AP et al., 2012 [23]), keeping 250–900 base long fragments, and filtering out those having more than 25% nucleotides with a Phred score ≤ 3. Quality filtering, taxonomy assignments, and diversity analyses of the samples were performed using the QIIME suite (release 1.9.0; [24]). After quality control, 3 samples out of 72 were found inappropriate for downstream analysis and thus removed from the data. Filtered reads were clustered into Operational Taxonomic Units (OTUs), using the uclust algorithm at 97% similarity. Taxonomic assignment was performed against the Greengenes 16S rRNA database (release 13_8, http://greengenes.microbio.me/greengenes_release/gg_13_8_otus, accessed on 6 December 2024) through the RDP classifier at 0.5 confidence [25]. Singletons (i.e., OTUs with only 1 supporting read) were discarded as likely chimeric sequences. To have comparable sequencing depths on all samples, OTU table was normalized to the least sequenced sample.

Alpha-diversity evaluation was estimated according to several microbial diversity metrics (chao1, Shannon index, observed species, and Faith’s phylogenetic distance) and β-diversity analysis was conducted using both weighted and unweighted UniFrac metrics [26] and through Principal Coordinate Analysis (PCoA).

### 2.5. Volatile Fatty Acid Analysis

The VFAs were determined by high-pressure liquid chromatography (HPLC, Perkin-Elmer, Boston, MA, USA, series 200; Chrompack organic acids column, n 28350, Middelburg, The Netherlands) at a wavelength of 210 nm. The injection was performed at 60 °C, with 0.8 mL/min of mobile phase flow. The mobile phase included 0.01 N H_2_SO_4_. The sample injection was performed with 50 µL of filtered sample. Peak detection was by reference to an external standard. The standards were run every 5 samples.

### 2.6. Determination of Enzymatic Activity Determination

Cellulase (EC 3.2.1.4), α-amylase (EC 3.2.1.1), and xylanase (EC 3.2.1.32) activities were determined through Radial Enzymatic Diffusion (RED) method [24], modified as described by [27] and expressed as area of the halo. The Petri dishes used for the assay contained 0.5, 0.5, or 0.1% (*w*/*v*) of cellulose (cellulose powder from cotton linter, code 22183, Fluka BioChemika, Buchs, Switzerland), starch (soluble starch, code 417585, Farmitalia Carlo Erba S.p.a, Milano, Italy), or xylan (AZCL-Arabinoxylan from wheat, cod. I-AZWAX, Megaxyme, Wicklow, Ireland) as substrates, respectively. Each substrate was solubilized with 1.5% (*w*/*v*) agar (Agar N1, code LP001, Oxoid, Milan, Italy) in a specific buffer, represented by 100 mM Na-acetate, pH 5.0 for cellulase; 100 mM Na-acetate, pH 4.8 for α-amylase; and 100 mM Na-citrate, pH 5.3 for xylanase. Gelation was obtained by heating at 100 °C for 12 min.

For each enzyme, the analysis was repeated in quadruplicate in 4 different Petri plates. In each Petri dish, 4 aluminum cylinders were placed to create the wells for the inoculation of RF to be tested. After cooling to 50 °C, 20 mL aliquots of gel were poured into the Petri dishes (90 mm diameter) while vigorously stirring, yielding a gel depth of 3 mm. After the agar gelation, the aluminum cylinders were removed to obtain the circular wells (diameter 10 mm) in the agar layer.

In each well, 300 μL of filtered RF were inoculated and then incubated for 16 h at 50 °C for cellulase and amylase testing and at 37 °C for xylanase testing. Cellulase and amylase hydrolysis were revealed by staining, obtained by flooding plates with 0.2% (*w*/*v*) I_2_ in 2.0% KI staining solution for 15–20 min or by Lugol solution diluted at 1:40 for a few seconds, respectively; both staining procedures were followed by multiple rinses with water. Xylan hydrolysis halos were already evident after incubation and thus no staining procedure was needed.

The halo of hydrolysis was measured by the MeazureTM 2.0 software (C Thing Software, Sunnyvale, CA, USA). The well’s area was then subtracted from the total area of the halo circle. Results were expressed as corrected area of the surface of the halo (mm^2^).

### 2.7. Statistical Analysis

Statistical evaluation of α-diversity indices was performed by non-parametric Monte Carlo-based tests through the QIIME pipeline, and β-diversity differences were assessed by a permutation test with pseudo-F-ratios using the “adonis” function from R package “vegan” (version 2.0-10). Alpha-diversity evaluation was represented in the form of a rarefaction curve, calculating the alpha-diversity metric on a subset of sequences extracted from the samples, ranging from 500 to 26,500 by steps of 500 sequences each. In this way, evaluating the reach of a plateau in the curves, it was possible to assess if the sequencing depth of the samples was sufficient to completely describe the ecosystem and, at the same time, check if the experimental classes were characterized by different biodiversity. Beta-diversity analysis, on the other hand, was represented by means of a Principal Coordinate Analysis (PCoA), which summarizes and attempts to represent inter-object dissimilarity in a low-dimensional, Euclidean space, taking a dissimilarity matrix (the UniFrac-based distance matrix for each pair of samples) as the input.

Data on performances and microbial abundance were analyzed through the GLM procedure of SAS software, version 9.4 (SAS Institute Inc., Cary, NC, USA) according to the following linear model:

y*_ijk_* = μ + cow*_i_* + time*_j_* + week*_k_* + (cow × time)*_ij_* + (cow × week)*_ik_* + e*_ijk_*,

where y*_ijk_* is the dependent variable (performances or microbial abundance); μ is the overall intercept of the model; cow*_i_* is the fixed effect of the *i*th cow (*i* = 1 to 4); time*_j_* is the fixed effect of the *j*th time after feeding (*j* = 0, 4, 8 h); week*_k_* is the fixed effect of the *k*th week of sampling (*k* = 1, 2, 3); (cow × time)*_ij_* is the fixed interaction effect between the *i*th cow and the *j*th time after feeding; (cow × week)*_ik_* is the fixed interaction effect between the *i*th cow and the *k*th week; and e*_ijk_* is the random residual ~N(0,σ^2^_e_), where σ^2^_e_ is the error variance. Data are presented as least squares means and standard error, and multiple comparisons of least squares means were performed using Bonferroni post hoc test (*p* < 0.05).

During model building, cow was tested as a random effect, considering repeated measures were available. While this approach is the preferred and appropriate one when dealing with repeated data, the covariance matrix in our study was not positive-definite, which underlines potential estimation issues. Therefore, we opted for including cow as fixed effect and for interactions with time and week in the model. Notably, the estimated least square means from the two models/approaches were consistent. Due to the limited sample size, the two models are nearly equivalent, and the variance from repeated measures of the same individual was not significant.

Moreover, Spearman’s correlations between microbes’ profiles at phyla, family, and genus level and rumen functional indicators (digestibility indices, enzymatic activities, and VFA) were tested and are displayed as heat map tables with their corresponding coefficient of correlation (r).

## 3. Results

### 3.1. pH and Total Tract Digestibility

Significant interactions were detected amongst the individual cows and intervals after feeding (*p* = 0.036), and the individual cows and sampling week (*p* = 0.029). The pH of RF were similar across daily (*p* = 0.051) intervals, showing a trend for the lowest pH observed at 4 h after feeding (Table 2). No differences were found between weekly intervals (*p* = 0.077), while some differences were observed among individual cows (*p* = 0.007), with DC3 and DC4 showing lower values than DC1.

The ttNDFDe depicted significant interactions amongst the individual cows and intervals after feeding (*p* = 0.005), and the individual cows and sampling weeks (*p* = 0.003) (Table S1). Differences were observed when data were analyzed using individual cows as a fixed factor, with DC3 showing lower values than DC2 and DC4 (*p* = 0.009; Table 2). The ttNDFDe was similar amongst the daily intervals, while a lower ttNDFDe was observed in the first sampling week compared to the second and third ones (*p* < 0.001; Table 2).

Regarding the ttaDMDe, no interactions were highlighted amongst the individual cows and intervals after feeding, and the individual cows and sampling weeks (Table S1). Alterations were observed only amongst the three consecutive sampling weeks (*p* = 0.002). In detail, the average ttNDFDe and ttaDMDe increased in weeks 2 and 3 compared to week 1 (Table 2). Overall, the in vivo digestibility values were similar with time after feeding, while differences were observed between sampling weeks (Table 2).

### 3.2. Volatile Fatty Acids

The interactions amongst the individual cows and intervals after feeding, and the individual cows and sampling weeks for the concentration of acetic, propionic, and butyric acid, were not significant (Table S1). Thus, no differences between DCs, times relative to feeding, and weekly intervals were observed for these VFAs in general. Only butyric acid was higher (*p* = 0.009) at T8 after feeding compared to T0 and T4 (Table 2).

Lactic acid showed a significant interaction (*p* < 0.001) between DCs and intervals after feeding (Table S1) since it was found to be higher in the DC1 and DC4 compared to DC3 (*p* = 0.007) and increased at T8 after feeding (*p* < 0.001; Table 2).

Overall, except for lactic acid, there were no substantial alterations in the principal acid production when the DC and sampling weeks were used as fixed effects (Table 2).

### 3.3. Enzymatic Activity

Concerning cellulase and xylanase activities, no interactions were observed amongst the individual cows and intervals after feeding, and the individual cows and sampling weeks, while amylase reported a significant interaction (*p* = 0.013) between DC and sampling week (Table S1). Xylanase activity was higher in DC3 compared to the other DCs (*p* < 0.001), and in DC4 compared to DC2 (Table 2). Xylanase progressively increased after feeding showing a difference at T8 (Table 2). Weekly variations were found in both amylase and cellulase with an opposite pattern (Table 2). More specifically, amylase was higher (*p* < 0.001) during the second week while cellulase was lower (*p* = 0.036; Table 2).

### 3.4. 16S rRNA Gene Amplicon Analysis

Illumina MiSeq sequencing resulted in 146,458 ± 71,341 reads for liquid and 152,847 ± 72,891 reads for solid rumen samples after amplicon rebuilding and quality filtering. In total, 29 different phylum-level OTUs, 182 family-level OTUs, and 239 genus-level OTUs were identified considering both liquid and solid fractions. Amongst the phylum level, Bacteroidetes (44.7%), Firmicutes (36.3%), Verrucomicrobia (3.3%), Proteobacteria (3.0%), Cyanobacteria (1.8%), and Fibrobacteres (1.8%) comprised 90.8% of the identified OTUs considering both fractions.

**Figure 1:**
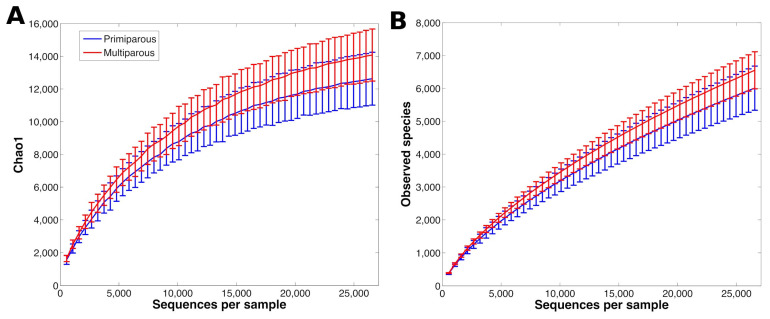
Rumen liquid bacterial rarefaction curve calculated according to chao1 (**A**) and observed species (**B**) metrics for all samples, representing the α-diversity difference between primiparous (DC1 and DC2) and multiparous (DC3 and DC4) cows. Curves were drawn using the least sequenced sample (~28,000 reads) as upper limit for the rarefactions.

**Figure 2:**
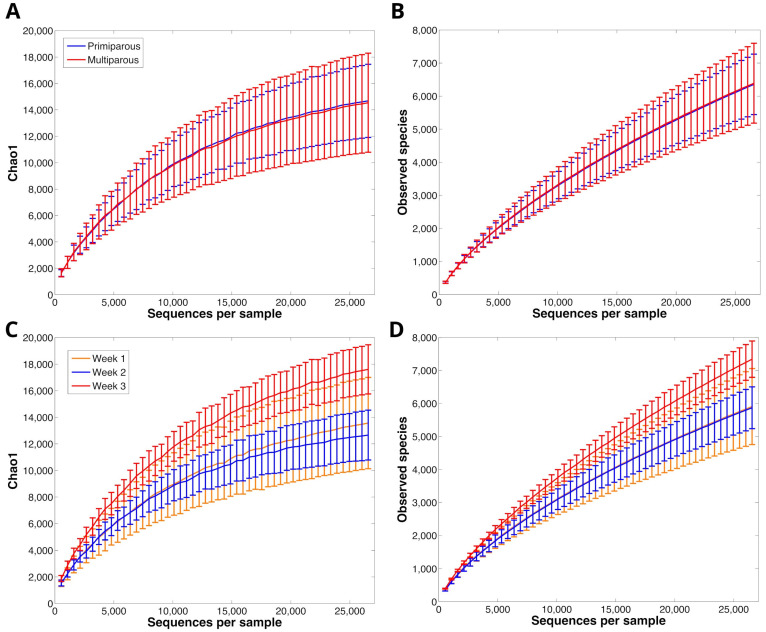
Rumen solid bacterial rarefaction curve calculated according to chao1 (**A**) and observed species (**B**) metrics, representing the α-diversity difference between primiparous (DC1 and DC2) and multiparous (DC3 and DC4) cows. Rarefaction curves calculated according to chao1 (**C**) and observed species (**D**) representing the α-diversity difference between three consecutive sampling weeks. Curves were drawn using the least sequenced sample (~28,000 reads) as upper limit for the rarefactions.

The rarefaction curve analysis, which assessed species richness, suggested that our sequencing depth was enough to sufficiently describe the main components of the rumen microbiota biodiversity. Good’s coverage was estimated in 0.85 ± 0.02 (range: 0.81–0.89) and 0.84 ± 0.03 (range: 0.80–0.93) for the liquid and solid fractions, respectively (Figure 1 and Figure 2). The α-diversity in the liquid fraction indicated that multiparous cows had a richer microbiota structure compared to primiparous cows (chao1, Shannon, and observed species metrics, *p* = 0.017, 0.010, and 0.022, respectively; Figure 1). The α-diversity in solid fraction indicated that the microbiota structure at week 3 of sampling was the richest during the observed period (chao1, Shannon, observed species metrics, and PD whole tree *p* < 0.05; Figure 2C).

**Figure 3:**
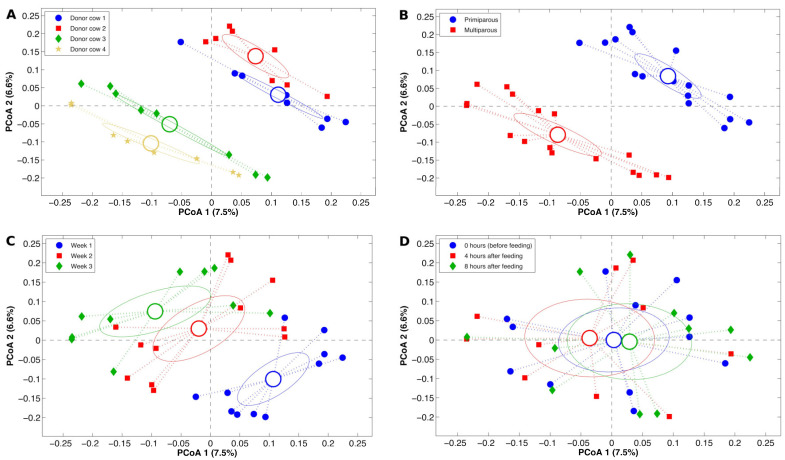
Principal Coordinates Analysis plots based on the unweighted UniFrac distances discriminate the four donor cows (DCs, (**A**)), the two parity groups (**B**), the three sampling weeks (**C**), and the three intervals between feeding (**D**) based on rumen liquid microbiota β-diversity. In the plots, each point represents a sample and is colored according to the experimental category. Centroids represent the average coordinate of all the points and ellipses are the SEM-based 95 confidence intervals. For each plot, the first and the second components of the PCoAs are presented.

**Figure 4:**
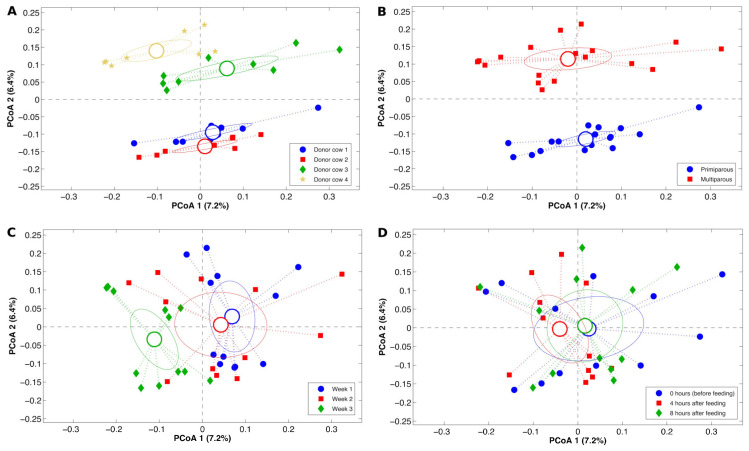
Principal Coordinates Analysis plots based on the unweighted UniFrac distances discriminate the four donor cows (DCs, (**A**)), the two parity groups (**B**), the three sampling weeks (**C**), and the three intervals between feeding (**D**) based on rumen solid microbiota beta diversity. In the plots, each point represents a sample and is colored according to the experimental category. Centroids represent the average coordinate of all the points and ellipses are the SEM-based 95 confidence intervals. For each plot, the first and the second components of the PCoAs are presented.

The β-diversity in liquid fraction based on the unweighted UniFrac distance (Figure 3) differed among the four DCs (*p* < 0.001), and we also observed a discrimination attributable to the parity of the DC (*p* < 0.001). Additionally, the microbiota diversity in liquid fraction showed an alteration (*p* < 0.001) between the sampling weeks but the variables were not discriminated based on the time after feeding (*p* = 0.975; Figure 3). Similarly, the β-diversity in the solid fraction, based on the unweighted UniFrac distance between the four DCs, was different (*p* < 0.001), and there was discrimination based on their parity number (*p* < 0.001). Additionally, the bacterial diversity in solid fraction showed an alteration (*p* < 0.001) between the sampling weeks but the variables were not discriminated, again based on the time after feeding (*p* = 0.792; Figure 4). Because rumen biochemistry variability is also dependent on low-abundant bacteria, the unweighted UniFrac distances (which include all the observations) was used to consider the impact of these components of the microbiota for inter-sample diversity.

Following the observations of the microbiota diversity in the liquid fraction, the two dominant phyla, *Bacteroidetes* and *Firmicutes*, were significantly altered amongst the three sampling weeks (Table 3, Figure S1). *Bacteroidetes* was higher during week 2 compared to week 1 (*p* = 0.006), while *Firmicutes* was higher in week 3 (*p* < 0.001). These changes in the dominant rumen phyla were not observed in the solid fraction (Table 4, Figure S2). Alterations were also found considering the sampling week for *Verrucomicrobia* (*p* = 0.001), *Proteobacteria* (*p* = 0.002), *Cyanobacteria* (*p* = 0.003), and *Fibrobacteres* (*p* = 0.032) in the liquid fraction, and *Cyanobacteria* (*p* < 0.001) and *Fibrobacteres* (*p* < 0.001) in the solid one (Table 3 and Table 4). At the phylum level, DC1 and DC2 showed a higher relative abundance of *Cyanobacteria* in liquid (*p* = 0.001) and solid (*p* = 0.001) fractions, respectively. Interestingly, the relative abundance of rumen phyla in both liquid and solid fractions was not affected by the time after feeding (Table 3 and Table 4).

The dominant rumen bacterial family was the *Prevotellaceae* without noteworthy alterations based on the investigated factors, in both liquid and solid fractions (Table 3 and Table 4). However, other *Unclassified (Uncl)_Bacteroidales* had a higher relative abundance in RF and solid phases of DC4 (*p* < 0.001 and *p* = 0.002, respectively). The relative abundance of *Lachnospiraceae* decreased at T4 and T8 compared to their abundance at T0 in the liquid fraction (*p* = 0.001; Table 3), while they were higher in the solid fraction of DC2 (*p* = 0.016; Table 4). *Uncl_Clostridiales* and *Ruminococcaceae* progressively increased from week 1 to week 3 only for the liquid fraction (*p* < 0.001). Bacteria belonging to *RF16* family were found at a lower relative abundance in the liquid fraction of DC3 and DC4 (*p* < 0.001). The relative abundance of *Fibrobacteraceae* rose during week 3 only in the solid fraction (*p* < 0.001; Table 4).

At the genus level, *Prevotella* was the dominant ruminal genus that was not affected by the considered factors in both fractions (Table 3 and Table 4). The relative abundance of *Succiniclasticum* was higher in the rumen liquid of DC2 (*p* = 0.040; Table 3). A progressive decrease in the relative abundance of *Anaeroplasma*, *Uncl_Veillonellaceae*, *Treponema*, and *Fibrobacter* was observed from week 1 to week 3 in the liquid fraction (*p* = 0.036, *p* = 0.013, *p* < 0.001, and *p* = 0.05, respectively; Table 3), while in the solid fraction, *Fibrobacter*, *Butyrivibrio*, and *Ruminococcus* had a higher relative abundance in week 3 (*p* = 0.001, *p* = 0.012, and *p* = 0.014, respectively; Table 4). On the contrary, the relative abundance of *Uncl_Paraprevotellaceae*, *Uncl_Ruminococcaceae*, and *Ruminococcus* progressively increased at the same intervals in the liquid fraction (*p* < 0.001, *p* < 0.001, and *p* = 0.050; Table 3).

The time after feeding did not alter the rumen microbiota structure, except for the relative abundance of *Clostridium* (*p* = 0.022 and *p* = 0.025 in liquid and solid fraction, respectively) and *Uncl_Lachnospiraceae* (*p* = 0.003 in liquid fraction), which decreased after feeding (Table 3 and Table 4).

### 3.5. Correlations Among the Tested Variables

**Figure 5:**
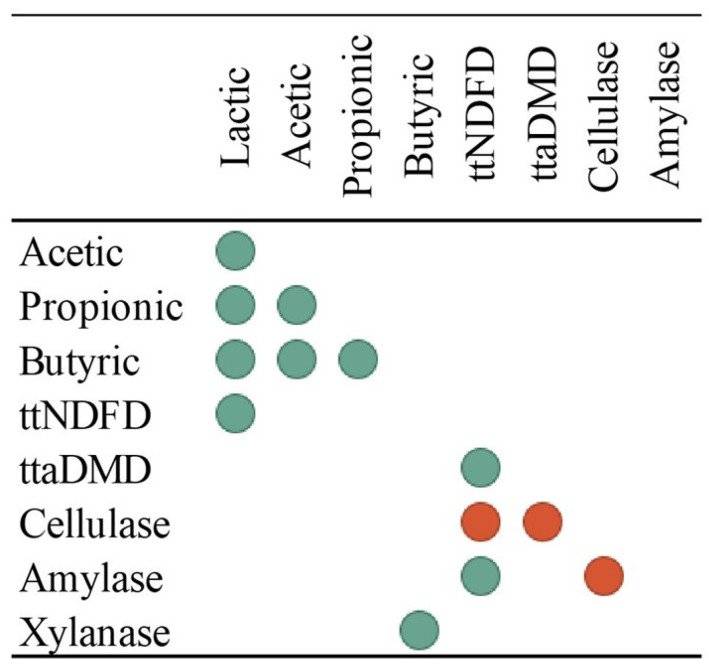
Spearman’s correlations of volatile acid concentration, enzymatic activities, total tract neutral detergent fiber digestibility (ttNDFDe), and total tract apparent dry matter digestibility (ttaDMDe) of the donor cows. The green dots indicate significant positive correlations, and the red dots indicate significant negative correlations.

Overall, the concentrations of VFA were positively correlated (*p* < 0.001), with the strongest correlation amongst butyric and acetic acids (r = 0.708) and between butyric and propionic acids (r = 0.868; Figure 5). The ttNDFDe was positively correlated with lactic acid (r = 0.415) and the ttaDMDe only (r = 0.418). No correlations were observed between VFA and EA except between butyric acid and xylanase activity (r = 0.365; Figure 5). Cellulase activity was negatively correlated (*p* < 0.010) with amylase activity (r = 0.311), ttaDMDe (r = 0.284), and ttNDFDe (r = 0.361). On the contrary, amylase activity was positively correlated (*p* < 0.010) with the ttNDFDe (r = 0.245; Figure 5).

The ttNDFDe and ttaDMDe were strongly correlated with the microbiota structure, with both positive and negative correlations in the liquid fraction and mainly positive correlations in the solid fraction (Table 5 and Table 6). More specifically, the ttaDMDe was positively correlated (*p* < 0.05) with Firmicutes, Bacteroidetes, and Actinobacteria at the phylum level; *Bacteroidales other*, *Coriobacteriaceae*, *Ruminococcaceae*, and *Uncl_Clostridiales* at the family level; and *Uncl_Paraprevotellaceae* and *Uncl_Ruminococcaceae* at the genus level in the liquid fraction. On the contrary, in the same fraction, negative correlations (*p* < 0.05) were found with Proteobacteria, Cyanobacteria, Spirochaetes, Tenericutes, TM7, and Lentisphaerae at the phylum level; *Uncl_ YS2*, *Spirochaetaceae*, *F16*, and *Victivallaceae* at the family level; and *Treponema* at the genus level. In a similar way, the ttNDFDe was positively correlated with *Uncl_Clostridiales* and *Ruminococcaceae* at the family level, while negative correlations were found with *Veillonellaceae*, *F16*, and *Victivallaceae* in the liquid fraction. Interestingly, all the correlations (except for the one with *Planococcaceae*) found in both the ttaDMDe and ttNDFDe with solid adhered bacteria were positive (Table 6).

Lactic acid was positively correlated (*p* < 0.05) with members of Firmicutes and Bacteroidetes phylum, while negative correlations (*p* < 0.05) were found with the *Lachnospiraceae* and *Clostridiaceae* families and *Butyrivibrio* genus in the liquid fraction (Table 5). In the solid fraction, lactic acid was negatively correlated (*p* < 0.05) with the *Anaeroplasmataceae* and *Clostridiaceae* families and *Treponema* genus (Table 6). Acetic acid showed weak correlations with rumen microbiota, reporting a negative correlation with *BS11* in liquid (*p* < 0.05) and a positive correlation with *Lachnospiraceae* in the solid fraction (*p* < 0.05; Table 5 and Table 6). Propionic acid was negatively correlated (*p* < 0.05) with *BS11* and positively correlated (*p* < 0.05) with *RF16* in the liquid fraction, while only a positive correlation (*p* < 0.05) with *Uncl_Lachnospiraceae* was found in the solid fraction. Butyric acid was negatively correlated (*p* < 0.05) with *Lachnospiraceae* and *Clostridiaceae* and consequently with *Butyrivibrio* and *Clostridium* in the liquid fraction (Table 5).

Interestingly, cellulase activity was positively correlated (*p* < 0.05) only with *Veillonellaceae* and *Succiniclasticum* in the liquid fraction, and xylanase activity was positively correlated (*p* < 0.05) with *BS11* and *Coriobacteriaceae*, and negatively correlated (*p* < 0.05) with *Lachnospiraceae* and *Clostridiaceae* in the liquid fraction (Table 5). Xylanase was strongly correlated with solid adhered bacteria since positive correlations (*p* < 0.05) were found with Actinobacteria, Bacteroidetes, *BS11*, *Coriobacteriaceae*, *Paraprevotellaceae*, *Prevotellaceae*, *Uncl_Veillonellaceae*, and *YRC22* while negative correlations (*p* < 0.05) were observed with Firmicutes, *Clostridiales other*, and *Lachnospiraceae other* (Table 6). Finally, amylase activity was strongly correlated with microbiota in the liquid fraction and negatively correlated (*p* < 0.05) with Firmicutes, *Ruminococcaceae*, *Uncl_Bacteroidales*, and *Uncl_ Prevotellaceae*, whilst positive correlations (*p* < 0.05) were found with the Fibrobacteres, Spirochaetes, and Tenericutes phyla; the *Anaeroplasmataceae*, *Fibrobacteraceae*, *Moraxellaceae* RF16 RFP12 *Spirochaetaceae* families; and the *Acinetobacter*, *Anaeroplasma*, *CF231 Fibrobacter*, and *Treponema* (Table 5).

## 4. Discussion

Despite the individual variations found in the pH of the RF, with 2 DCs showing lower pH values compared to one of the primiparous cows enrolled in this study, the pH values found are, as expected, within physiological ranges (6.2–6.8) in the reticolo-rumen [28], typical of hay-based diets [29]. Therefore, the pH can be considered, in this specific case, a factor that did not affect the other parameters studied.

Except for the ttNDFDe of the DC3, there were no variations in the ttNDFDe and ttaDMDe between the 4 DCs that also showed a consistent digestibility among intervals after feeding. Moreover, both ttNDFDe and ttaDMDe increased overall during the second and third sampling weeks, matching the differences in the liquid and solid phase microbiota composition highlighted by the weekly β-diversity. Indeed, the tight linkage between the rumen bacteriome’s structure and digestibility indices was also highlighted by the correlations of the present study. Consistently, the relation between the microbiota and animals’ performance has been well summarized in the literature [30]. Microbial VFAs can in fact contribute more than 80% to the total host metabolizable energy as demonstrated in sheep [31]. In our study, the average acetate–propionate–butyrate molar proportion was 73:16:10, which is comparable to the proportions observed in the rumen of cows fed high-forage diets [32]. However, the VFA production did not follow the weekly trend observed in the microbiota and digestibility indices, since all the organic acids considered were constant across the weeks. Concerning the enzymatic activity, xylanase seems to follow the trends observed for digestibility and microbiota, with increments in the second and third weeks, while cellulase roughly showed an opposite trend. On the other hand, amylase appeared to have inconsistent variations when related to the other parameters. This partial inconsistency between rumen bacteria and their functional biomarkers (digestibility, enzymatic activities, and VFA production) could be explained by the observations that rumen microbes having different taxonomic compositions can show identical metabolic functions [33], suggesting that different bacteria encode for the same functions and that a difference in the microbiota at the taxonomic level may not be directly associated with the metabolic functions that affect the host.

Regarding the correlations between the digestibility indices and the rumen microbiota, ttaDMDe was positively correlated with the Firmicutes phylum and especially with the *Clostridiales* and *Ruminococcaceae* families. These findings were not confirmed in the solid fractions where the Firmicutes phylum was positively related only to the ttNDFDe. These correlations also reflect the biological relationship between the considered variables, since the Firmicutes taxa (which include the *Ruminoccoaceae* family) encode most of the cellulose- and hemi-cellulose-degrading enzymes within the rumen [34]. A deeper assessment of the whole rumen microbiota, including the eukaryotes and especially anaerobic fungi and protozoa (not evaluated in the present study [35]), could explain this inconsistency. In detail, it has been demonstrated that both ruminal protozoa and anaerobic fungi [36] exert strong fibrolytic activity since they also encode a wide range of CAZyme genes. Nevertheless, in our study, the contribution of protozoa in the rumen of grass-fed cows was expected to be negligible since both their abundance and diversity are reduced in high-forage diets [37,38]. On the contrary, it has been reported that the abundance of anaerobic fungi is usually increased in the rumen with a low-grain diet [39], leading us to the assumption that the anaerobic fungi diversity and abundance may have been the link between the rumen microbiota and ttNDFDe. Lastly, the lack of positive correlation between the rumen fibrolytic taxa and ttNDFDe may be a result of the limitations of the inoculum collection technique adopted [4] and the partial digestion of dietary fiber by the intestinal microbes [5]. This process cannot be measured based on our dataset but could have masked the differences in ruminal digestion through a post-ruminal partial compensatory effect [40]. As recalled in the literature [41], in fact, the lower tract digestion of cellulose and hemicellulose ranged from 18.5 to 49.5% and 2.5 to 46.0%, respectively, and a value of post-ruminal true digestibility of 20% is considered as a reasonable average. Thus, the evaluation of the ttaDMDe and of the ttNDFDe should be considered only as a rough evaluation of the rumen’s digestive capacity.

From the evaluation of the rumen microbiota in terms of biodiversity and composition, a variation related to the parity of the animals (with primiparous cows having a lower richness) appears. A similar age effect on the rumen microbiota was also described in the literature [42], in which diversity increased with age, and in another study where multiparous cows showed higher rumen microbiota diversity [43]. Although there were no alterations of the ttaDMDe and only partial variations in the ttNDFDe digestibility indices amongst the DCs, the richness and diversity of the ruminal bacterial microbiota are important indicators of the rumen’s degradative activity and consequently host efficiency. It has been observed that the microbiota of less efficient cows have been characterized as more diverse in taxonomic composition, employing a higher number of metabolic pathways than the microbiota of more efficient cows [44]. Conversely, efficient cows’ microbial communities generally have more dominant taxa and rely on a smaller number of metabolic pathways that are more energetically valuable to the animal [44]. Diversity, therefore, might be negatively associated with the microbe’s ability to supply its host with the energy needed for production [30]. However, in our study and the reported literature, microbiome diversity was assessed in a single region of the 16S. Summarizing the above, as parity may affect the rumen diversity and metabolic functions, variations might also have occurred in the overall RF degradative capacity. Beyond the understanding of rumen biochemistry, in the realms of our more applicable objective, the cows’ parity could also be another factor to be considered during the collection of RF as inoculum for in vitro NDFD trials. In practice, this information could help minimize the possible impact of microbial diversity fluctuations to avoid inter-assay variabilities and dissimilar outcomes between different incubation batches.

The time intervals relative to feeding did not substantially affect the rumen microbiota structure, as observed by the PCoA and the negligible individual changes. This outcome is consistent with the literature [45], where the individual differences in the bacteria composition had a greater impact than the daily sampling time. In our study, only *Lachnospiraceae* members, namely *Butyrivibrio* and *Pseudobutyrivibrio*, showed a reduction in the time points following feed supply. It could be hypothesized that the rumen microbiota structure and, consequently, its metabolism was not sharply affected by the feed administration. More specifically, the hay-based diet rich in NDF and low in N and starch minimized the availability of rapidly fermented substrates (fast pool) for rumen microbes, resulting in a more constant metabolic activity. Additionally, the slower intake of the hay diet compared to the TMR diets reported in the literature—due to the lower palatability and higher volume per nutrient units—prolongs the feed consumption during the day, also leading to a more stable rumen function. In this light, several authors reported that cows fed a low-concentrate diet had 68% more frequent visits per day to the feeders with 30% longer duration per visit and a lower DMI [46]. Other authors also reported that cows fed with an 80:20 forage-to-concentrate (F:C) ratio spent more than double the time per day in feeders compared to those fed with a 20:80 F:C ratio [47]. Beyond the unaffected rumen composition, VFA production was also found to be identical pre- and post-feeding (T0 and T4, respectively), with an increase in butyrate and lactate only 8 h after feeding. Similarly, in the cited study [47], the concentrations of acetate, propionate, and butyrate were not considerably affected pre- and post-feeding (0, 3, and 6 h post-feeding) in the rumen of cows fed the diet at an 80:20 F:C ratio, whereas they were substantially increased after feeding in the rumen of high grain-fed cows. Although most of the investigated variables were not affected, the rumen enzymatic potential depicted some numerical fluctuations at an individual level. These fluctuations did not follow a certain trend within each enzyme; thus, an overall statistical analysis of enzymatic potential from all four DCs indicated that there was no effect from the daily sampling time except for the case of xylanase, which increased at T8 after feeding, consistent with butyric acid [48].

On the other hand, pooling the data concerning the RF from the four DCs was not able to fully balance the EA across the sampling weeks. In fact, the cellulase activity persistently tended to decrease during the second and third weeks overall, while amylase activity increased in the second week. As reported in the literature [49], ambient temperature is among the factors influencing the absorption of volatile fatty acids by the rumen. Moreover, ambient temperature influenced animal behavior, drink and meal bouts and sizes, and rumination time [50]. Thus, it can be hypothesized that behavioral changes and variations in VFA accumulations within the rumen may lead to fluctuations in ruminal pH, potentially promoting certain enzymatic activities while suppressing others, or altering nutrient digestibility. Although the seasonal and sampling time alteration in both rumen microbial structure and fermentation has been previously reported [14,15], their impact has never been studied as a function of the degradative capacity of the inoculum. Interestingly, the suppression of cellulase activity was accompanied by a rise in amylase activity, indicating a biological response of the rumen to preserve its overall degradative capacity. In this scenario, it is of high importance to assess if different CAZymes profiles with similar overall degradative potential could influence the repeatability of in vitro digestion outcomes. Moreover, this study primarily focused on ruminal inoculum collected via an esophageal probe, which does not include the larger feed particles of the rumen mat where most of the fiber degradation occurs. Thus, this sampling method may give different enzymatic results compared to others. According to the literature [51] despite no differences being detected in the microbiome of rumen fluid collected by an oro-esophageal probe or rumen fistula, the metabolome composition of the fluid and particulate samples were different in both collection techniques. However, the filtration protocol adopted in this study is recognized worldwide for digestibility analyses and adopted independently from the inocula collection method.

The above observations seem to confirm the importance of running in vitro digestibility trials using rumen inocula collected at different intervals (e.g., days or weeks), possibly reducing the errors in in vitro assays.

Assuming that the enzymatic profile of RF can be considered a qualitative reflection of the ruminal degradative potential [52] and a potential measure of its degradative capacity [53,54], it constitutes the principal factor that should be addressed to evaluate the properness of RF as an inoculum for in vitro NDFD trials. Considering the aforementioned outcomes, the collection of RF as an inoculum for in vitro digestibility trials from hay-fed cows offers several advantages that could minimize the variability of the process and strengthen the repeatability of the results since we found a similar microbiota structure and metabolic activity pre- and post-feeding. This stability possesses pivotal importance and can ensure the activity of the inoculum even when RF is collected at slaughtering if cattle are fed forage ad libitum. In this case, where the intervals of the last feeding are unknown, the presence of stable rumen metabolic activity is in fact crucial. It should be noted here that collecting RF from culled animals at slaughtering is encouraged since the rumen fluid is a waste product for the slaughterhouse and it prevents an invasive procedure from being performed on live animals, thus representing a more ethical and acceptable approach [2].

## 5. Conclusions

The results of this study showed limited variability in the ruminal microbiota of the dry hay-fed DCs at the time after feeding and moderate variations in individual animals, while major variations were observed when the weekly interval was considered. These sets of evidence validate previous findings about the best strategies to improve the fermentative consistency of RF as an inoculum, including pooling RF from multiple donor animals, and indicate the need to run digestibility trials at different intervals (e.g., days or weeks) to obtain experimental replicates. Additionally, this study provides new insights into the effect of individuals on the inoculum’s degradative potential through changes in the taxonomical level and how a hay-based diet can eliminate the within-day metabolic fluctuations at both the VFA and EA levels. Further studies should be carried out to assess the impact of the aforementioned recommendations on ivNDFD trials.

## Figures and Tables

**Table 1:** Chemical composition of the hay used to feed the donor cows.

Item ^a^	Mean	SD
DM, % as fed	85.0	2.90
Ash, % DM	8.71	0.84
Crude protein, % DM	9.93	1.22
Ether extract, % DM	1.56	0.06
NFC, % DM	18.6	3.33
aNDF, % DM	61.2	2.20
ADF, % DM	39.7	4.52
Lignin, % DM	7.93	2.78
Hemicellulose, % DM	21.5	3.17
Cellulose, % DM	31.8	2.10
NDFD 24 h, % DM	33.1	6.09

^a^ NFC = non-fiber carbohydrate; aNDF = amylase-treated neutral detergent fiber expressed as inclusive of residual ash; ADF = acid detergent fiber expressed as inclusive of residual ash; NDFD 24 h = in vitro aNDF digestibility at 24 h of fermentation.

**Table 2:** pH, total tract neutral detergent fiber digestibility (ttNDFDe, % DM), apparent dry matter digestibility (ttaDMDe, % DM), concentrations of acetic, propionic, butyric, and lactic acid (mg/100 mL), and enzymatic activities of amylase, cellulase, and xylanase (expressed as area of hydrolysis of the rumen fluids) in rumen fluid of the four donor cows (DC) at 0 h (before feeding), 4 h, and 8 h after feeding over three consecutive sampling weeks.

	Cow					Time After Feeding (h)				Week				*p*-Values		
	DC1	DC2	DC3	DC4	SEM	0	4	8	SEM	1	2	3	SEM	C	T	W
pH	6.82 ^a^	6.82 ^ab^	6.69 ^b^	6.69 ^b^	0.03	6.74	6.71	6.81	0.03	6.79	6.78	6.70	0.03	0.007	0.051	0.077
**Digestibility indices** (% DM)																
ttNDFDe	44.0 ^ab^	45.0 ^a^	41.7 ^b^	45.8 ^a^	0.75	44.6	44.1	43.7	0.65	39.3 ^b^	47.7 ^a^	45.4 ^a^	0.65	0.009	0.640	<0.001
ttaDMDe	73.9	75.1	74.5	75.3	0.46	74.2	74.7	75.2	0.40	73.3 ^b^	75.2 ^a^	75.6 ^a^	0.40	0.178	0.195	0.002
**Volatile fatty acid concentration** (mg/100 mL)																
Acetic acid	426.7	419.7	405.5	349.1	33.57	413.7	383.8	403.2	29.1	385.1	418.7	396.9	29.07	0.377	0.765	0.714
Propionic acid	92.3	93.9	94.7	81.5	8.49	88.1	80.8	102.9	7.36	86.3	92.3	93.2	7.36	0.673	0.130	0.770
Butiric acid	63.0	55.2	59.9	46.7	4.61	48.4 ^b^	52.0 ^b^	68.1 ^a^	3.99	55.1	58.9	54.5	3.99	0.115	0.009	0.711
Lactic acid	11.98 ^a^	8.13 ^ab^	5.39 ^b^	13.30 ^a^	1.29	4.52 ^b^	8.59 ^b^	15.99 ^a^	1.22	11.03	10.20	7.87	1.22	0.007	<0.001	0.215
**Enzymatic activity** (mm^2^)																
Amylase	116.1	140.0	135.7	134.6	6.39	129.1	127.1	138.7	5.54	130.5 ^b^	153.5 ^a^	110.8 ^b^	5.54	0.076	0.312	0.001
Cellulase	253.2	219.5	238.9	188.6	17.63	240.9	230.6	203.6	15.27	253.1 ^a^	191.9 ^b^	230.3 ^ab^	15.27	0.095	0.234	0.036
Xylanase	132.6 ^cb^	101.8 ^c^	176.2 ^a^	137.8 ^b^	8.23	120.9 ^b^	139.8 ^ab^	150.5 ^a^	7.13	121.7 ^b^	141.4 ^a^	148.2 ^a^	7.13	<0.001	0.030	0.046

Means with different superscript letters (a, b, c) in each row indicate significant differences (*p* ≤ 0.05) between donor cows, daily time intervals, and sampling weeks.

**Table 3:** Changes in microbiota relative abundances (% of the identified OTUs) in the rumen liquid fraction of the four donor cows (DC) at 0 h (before feeding), 4 h, and 8 h after feeding over three consecutive sampling weeks.

	Cow					Time After Feeding (h)				Week					*p*-Values	
	DC1	DC2	DC3	DC4	SEM	0	4	8	SEM	1	2	3	SEM	C	T	W
**Phylum**																
Bacteroidetes	48.86	45.31	47.44	48.44	1.00	46.94	46.55	49.05	1.00	45.1 ^b^	50.1 ^a^	47.4 ^ab^	1.00	0.1540	0.1736	0.0063
Firmicutes	26.85	29.86	29.00	29.02	1.63	29.4	31.03	25.62	1.48	24.3 ^b^	26.4 ^b^	35.4 ^a^	1.48	0.6134	0.0477	0.0002
Proteobacteria	1.3 ^ab^	2.2 ^a^	1.3 ^ab^	1.2 ^b^	0.20	1.26	1.69	1.6	0.19	2.2 ^a^	1.1 ^b^	1.2 ^b^	0.20	0.0227	0.2616	0.0017
Cyanobacteria	5.8 ^a^	2.7 ^b^	2.6 ^b^	2.9 ^b^	0.50	3.25	3.16	4.05	0.42	4.8 ^a^	3.4 ^ab^	2.2 ^b^	0.38	0.0014	0.2912	0.0030
Fibrobacteres	2.04	2.32	3.24	1.89	0.39	2.1	2.03	2.99	0.37	2.8 ^a^	2.8 ^a^	1.4 ^b^	0.40	0.1585	0.1631	0.0324
Verrucomicrobia	5.15	5.98	5.5	5.03	0.36	5.47	4.82	5.95	0.36	6.1 ^a^	6.1 ^a^	4.0 ^b^	0.36	0.3449	0.1100	0.0011
**Family**																
*Anaeroplasmataceae*	1.15	1.88	1.77	1.88	0.24	1.92	1.39	1.71	0.24	2.08 ^a^	1.79 ^ab^	1.15 ^b^	0.20	0.1966	0.2843	0.0430
*Bacteroidales other*	0.57 ^ab^	0.30 ^b^	0.57 ^ab^	0.64 ^a^	0.06	0.48	0.59	0.49	0.06	0.30 ^b^	0.59 ^a^	0.67 ^a^	0.10	0.0189	0.4438	0.0018
*BS11*	1.67 ^b^	0.51 ^c^	1.57 ^b^	2.88 ^a^	0.15	1.78	1.73	1.47	0.14	1.25 ^b^	1.72 ^ab^	2.01 ^a^	0.10	<0.0001	0.2873	0.0097
*Clostridiaceae*	1.00	1.39	0.85	0.92	0.12	1.16 ^ab^	1.22 ^a^	0.75 ^b^	0.12	1.09	0.91	1.13	0.12	0.0550	0.0238	0.3545
*Coriobacteriaceae*	0.44 ^b^	0.36 ^b^	0.77 ^b^	1.40 ^a^	0.09	0.63	0.82	0.79	0.09	0.52 ^b^	0.62 ^b^	1.09 ^a^	0.10	<0.0001	0.2251	0.0008
*Erysipelotrichaceae*	1.55	1.14	1.40	1.27	0.13	1.19	1.5	1.33	0.13	1.21	1.49	1.32	0.13	0.2535	0.2183	0.2771
*F16*	1.00	1.41	0.99	0.90	0.16	1.37	0.98	0.88	0.15	1.51 ^a^	0.84 ^b^	0.88 ^b^	0.15	0.2069	0.0653	0.0107
*Fibrobacteraceae*	2.04	2.31	3.24	1.88	0.39	2.10	2.02	2.98	0.39	2.84	2.83	1.43	0.39	0.1577	0.1647	0.0500
*Lachnospiraceae*	6.15	7.40	7.37	6.98	0.61	8.90 ^a^	6.24 ^b^	5.80 ^b^	0.51	7.57	5.85	7.51	0.51	0.4581	0.0012	0.0500
*Prevotellaceae*	23.55	24.41	26.04	24.24	0.82	24.83	23.10	25.75	0.69	24.69	25.4	23.59	0.69	0.2249	0.0631	0.2205
*RF16*	4.20 ^a^	4.92 ^a^	2.17 ^b^	2.04 ^b^	0.37	2.53 ^b^	3.71 ^ab^	3.76 ^a^	0.31	3.08 ^b^	4.47 ^a^	2.44 ^b^	0.31	<0.0001	0.0182	0.0012
*RFP12*	4.45	5.26	4.90	4.08	0.36	4.67	4.05	5.30	0.34	5.73 ^a^	5.21 ^a^	3.08 ^b^	0.34	0.1870	0.0747	0.0003
*Ruminococcaceae*	6.70	5.63	6.87	6.27	0.39	6.24	6.82	6.04	0.33	4.21 ^c^	6.16 ^b^	8.74 ^a^	0.33	0.1662	0.2873	<0.0001
*Spirochaetaceae*	1.28	1.40	1.45	1.22	0.15	1.50	1.07	1.45	0.14	2.08 ^a^	1.25 ^b^	0.69 ^c^	0.14	0.7301	0.1116	<0.0001
*Uncl_Bacteroidales* ^1^	13.15 ^ab^	9.64 ^c^	10.77 ^bc^	13.93 ^a^	0.58	11.84	12.01	11.77	0.54	10.83	11.84	12.94	0.54	0.0009	0.9544	0.0603
*Uncl_Clostridiales*	6.97	5.91	7.92	8.47	0.58	6.52	8.59	6.83	0.55	4.56 ^c^	7.54 ^b^	9.85 ^a^	0.55	0.0564	0.0500	<0.0001
*Uncl_SR1*	0.63 ^ab^	0.39 ^c^	0.75 ^a^	0.52 ^bc^	0.05	0.61	0.53	0.57	0.04	0.30 ^b^	0.64 ^a^	0.78 ^a^	0.04	0.0014	0.4649	<0.0001
*Veillonellaceae*	2.07	3.42	2.52	2.81	0.36	2.74	2.55	2.83	0.31	3.50 ^a^	2.14 ^b^	2.48 ^ab^	0.31	0.1126	0.8276	0.0186
*Victivallaceae*	1.51	2.28	2.03	1.82	0.25	2.24	1.76	1.75	0.24	3.72 ^a^	1.44 ^b^	0.58 ^b^	0.24	0.2943	0.2478	<0.0001
**Genera**																
*Anaeroplasma*	0.79	1.57	1.48	1.57	0.20	1.60	1.08	1.38	0.20	1.73 ^a^	1.43 ^ab^	0.90 ^b^	0.20	0.0796	0.2054	0.0357
*Butyrivibrio*	0.91	1.04	1.41	1.18	0.16	1.49	0.99	0.93	0.16	1.15	0.89	1.36	0.16	0.2798	0.0500	0.1216
*CF231*	2.49	2.56	1.74	1.86	0.17	1.91	2.10	2.46	0.17	2.59 ^a^	2.31 ^a^	1.58 ^b^	0.17	0.0500	0.0727	0.0028
*Clostridium*	0.96	1.17	0.85	0.84	0.11	1.12 ^a^	1.06 ^ab^	0.69 ^b^	0.11	0.94	0.89	1.04	0.11	0.2249	0.0216	0.5945
*Coprococcus*	0.36 ^b^	0.60 ^a^	0.53 ^ab^	0.45 ^ab^	0.05	0.56	0.47	0.42	0.04	0.53 ^ab^	0.38 ^b^	0.55 ^a^	0.04	0.0157	0.0663	0.0166
*Fibrobacter*	2.04	2.31	3.24	1.88	0.39	2.10	2.02	2.98	0.39	2.84	2.83	1.43	0.39	0.1583	0.1646	0.0500
*Lachnospiraceae other*	0.73	0.90	0.93	1.01	0.08	1.07	0.83	0.78	0.08	0.96	0.79	0.92	0.08	0.2334	0.0500	0.2518
*Prevotella*	23.09	23.99	25.45	23.54	0.78	24.30	22.54	25.21	0.73	24.41	24.84	22.79	0.73	0.2833	0.0755	0.1648
*Ruminococcus*	0.70 ^ab^	0.61 ^b^	0.88 ^a^	0.81 ^ab^	0.05	0.81	0.71	0.73	0.05	0.68 ^b^	0.55 ^b^	1.02 ^a^	0.05	0.0230	0.3216	<0.0001
*Succiniclasticum*	1.33 ^b^	2.48 ^a^	1.56 ^ab^	1.86 ^ab^	0.26	1.70	1.76	1.96	0.22	2.17	1.36	1.88	0.22	0.0405	0.6807	0.0500
*Treponema*	1.24	1.35	1.38	1.18	0.16	1.44	1.02	1.40	0.14	2.03 ^a^	1.19 ^b^	0.65 ^c^	0.14	0.7651	0.1067	<0.0001
*Uncl_BS11*	1.67 ^b^	0.51 ^c^	1.57 ^b^	2.88 ^a^	0.15	1.78	1.73	1.47	0.14	1.25 ^b^	1.72 ^ab^	2.01 ^a^	0.14	<0.0001	0.2873	0.0097
*Uncl_Lachnospiraceae*	3.04	3.41	3.27	3.11	0.27	4.05 ^a^	2.91 ^b^	2.66 ^b^	0.27	3.43	2.70	3.49	0.27	0.8213	0.0032	0.0722
*Uncl_Paraprevotellaceae*	1.31 ^a^	1.02 ^ab^	1.02 ^ab^	0.77 ^b^	0.09	0.93	1.04	1.13	0.08	0.62 ^c^	1.07 ^b^	1.40 ^a^	0.08	0.0058	0.1972	<0.0001
*Uncl_RFP12*	4.45	5.26	4.90	4.08	0.36	4.67	4.05	5.30	0.34	5.73 ^a^	5.21 ^a^	3.08 ^b^	0.34	0.1870	0.0747	0.0003
*Uncl_Ruminococcaceae*	5.16	4.40	5.49	4.93	0.35	4.84	5.54	4.62	0.30	2.94 ^c^	4.97 ^b^	7.08 ^a^	0.30	0.2141	0.1306	<0.0001
*Uncl_Veillonellaceae*	0.48	0.65	0.78	0.74	0.16	0.78	0.58	0.63	0.13	1.04 ^a^	0.56 ^ab^	0.39 ^b^	0.13	0.5620	0.5374	0.0135

^1^ Uncl: unclassified. Means with different superscript letters (a, b, c) in each row indicate significant differences (*p* ≤ 0.05) between donor cows, daily time intervals, and sampling weeks.

**Table 4:** Changes in microbiota relative abundances (% of the identified OTUs) in the rumen solid fraction of the four donor cows (DC) at 0 h (before feeding), 4 h, and 8 h after feeding over three consecutive sampling weeks.

	Cow					Time After Feeding (h)				Week					*p*-Values	
	DC1	DC2	DC3	DC4	SEM	0	4	8	SEM	1	2	3	SEM	C	T	W
**Phylum**																
Bacteroidetes	41.0	39.0	44.0	43.0	2.00	39.0	44.0	42.0	1.00	39.0	41.0	44.0	1.00	0.2739	0.0482	0.0686
Firmicutes	44.0	47.0	41.0	43.0	2.00	45.0	44.0	43.0	2.00	45.0	44.0	43.0	2.00	0.3144	0.6857	0.6580
Proteobacteria	4.60	4.20	5.50	3.40	2.80	6.80	1.30	5.10	2.50	6.90	5.20	1.10	2.50	0.9609	0.3036	0.2596
Cyanobacteria	0.27 ^ab^	0.32 ^a^	0.09 ^c^	0.15 ^bc^	0.03	0.18	0.24	0.21	0.03	0.03 ^c^	0.16 ^b^	0.44 ^a^	0.03	0.0005	0.4615	<0.0001
Fibrobacteres	1.17	1.23	1.46	0.78	0.19	1.30	1.40	0.80	0.16	0.66 ^b^	1.04 ^b^	1.78 ^a^	0.16	0.1242	0.0459	0.0006
Verrucomicrobia	1.40	1.40	0.90	1.40	0.20	1.00 ^b^	1.60 ^a^	1.20 ^ab^	0.20	1.10	1.40	1.40	0.20	0.2249	0.0441	0.4092
**Family**																
*Anaeroplasmataceae*	0.04 ^b^	0.10 ^ab^	0.12 ^a^	0.06 ^ab^	0.02	0.09	0.09	0.07	0.01	0.06	0.07	0.11	0.01	0.0203	0.5942	0.1077
*Bacteroidales other*	0.87 ^b^	0.89 ^b^	1.07 ^ab^	1.25 ^a^	0.07	0.89 ^b^	1.13 ^a^	1.04 ^ab^	0.07	0.80 ^b^	1.02 ^ab^	1.24 ^a^	0.07	0.0048	0.0430	0.0006
*BS11*	2.40 ^a^	1.16 ^b^	2.01 ^ab^	2.62 ^a^	0.29	1.76	2.45	1.94	0.25	2.36	2.13	1.66	0.25	0.0121	0.1627	0.1610
*Clostridiaceae*	2.01	2.40	2.14	2.14	0.19	2.57 ^a^	2.08 ^ab^	1.87 ^b^	0.19	2.18	2.25	2.09	0.19	0.5677	0.0248	0.8020
*Clostridiales other*	1.52 ^ab^	2.10 ^a^	1.40 ^b^	1.95 ^ab^	0.16	1.75	1.88	1.59	0.14	1.70	1.60	1.92	0.14	0.0181	0.3502	0.2562
*Coriobacteriaceae*	0.70 ^b^	0.63 ^b^	0.84 ^b^	1.50 ^a^	0.12	0.65 ^b^	0.93 ^ab^	1.18 ^a^	0.12	0.83 ^ab^	0.78 ^b^	1.16 ^a^	0.12	0.0002	0.0064	0.0295
*Erysipelotrichaceae*	0.45 ^a^	0.42 ^ab^	0.29 ^b^	0.39 ^ab^	0.04	0.35	0.43	0.39	0.03	0.45	0.34	0.37	0.03	0.0366	0.2350	0.0624
*F16*	1.32	0.97	0.92	1.10	0.17	0.83	1.23	1.17	0.14	1.28	0.91	1.04	0.14	0.3624	0.1393	0.2089
*Fibrobacteraceae*	1.17	1.22	1.45	0.77	0.19	1.27	1.40	0.80	0.16	0.65 ^b^	1.03 ^b^	1.78 ^a^	0.16	0.1222	0.0500	0.0006
*Lachnospiraceae*	13.55 ^ab^	14.57 ^a^	11.48 ^ab^	10.54 ^b^	0.86	13.20	12.13	12.27	0.74	11.13	13.22	13.25	0.74	0.0162	0.5553	0.0998
*Prevotellaceae*	18.84	17.65	22.25	18.48	1.21	17.42	19.63	20.87	1.05	19.16	18.15	20.62	1.05	0.0729	0.0920	0.2744
*RF16*	0.16	0.12	0.13	0.06	0.02	0.13	0.12	0.11	0.02	0.07	0.15	0.13	0.02	0.0746	0.7684	0.0522
*RFP12*	0.74	0.68	0.52	0.63	0.10	0.54	0.82	0.57	0.09	0.45 ^b^	0.69 ^ab^	0.80 ^b^	0.09	0.4777	0.0620	0.0318
*Ruminococcaceae*	8.40	9.05	9.02	10.65	0.98	8.34	10.35	9.15	0.85	10.72	8.70	8.41	0.85	0.4348	0.2722	0.1441
*Spirochaetaceae*	1.17	1.28	1.28	1.18	0.16	1.72 ^a^	1.13 ^b^	0.83 ^b^	0.14	0.96	1.28	1.44	0.14	0.9293	0.0011	0.0796
*Uncl_Bacteroidales* ^1^	13.64 ^ab^	14.70 ^a^	12.40 ^b^	15.75 ^a^	0.52	13.8 ^ab^	15.27 ^a^	13.28 ^b^	0.52	12.36 ^b^	14.91 ^a^	15.09 ^a^	0.52	0.0023	0.0180	0.0009
*Uncl_Clostridiales*	11.00	9.26	8.41	10.37	0.72	9.71	9.69	9.88	0.62	11.19 ^a^	8.81 ^ab^	9.27 ^b^	0.62	0.0889	0.9716	0.0360
*Unclassified_YS2*	0.27 ^ab^	0.31 ^a^	0.09 ^c^	0.14 ^bc^	0.03	0.18	0.23	0.20	0.03	0.02 ^c^	0.16 ^b^	0.42 ^a^	0.03	0.0005	0.4721	<0.0001
*Veillonellaceae*	3.72	5.02	4.69	3.76	0.46	4.52	4.33	4.03	0.40	1.98 ^b^	5.27 ^a^	5.63 ^a^	0.40	0.1507	0.6891	<0.0001
*Victivallaceae*	0.05	0.07	0.07	0.06	0.01	0.05	0.08	0.06	0.01	0.04	0.06	0.08	0.01	0.9142	0.1655	0.0939
**Genera**																
*Anaeroplasma*	0.03 ^b^	0.09 ^ab^	0.11 ^a^	0.06 ^ab^	0.02	0.08	0.08	0.06	0.01	0.05	0.07	0.09	0.01	0.0164	0.5345	0.1382
*Butyrivibrio*	1.74	1.81	1.50	1.35	0.14	1.64	1.56	1.60	0.14	1.32 ^b^	1.56 ^ab^	1.92 ^a^	0.14	0.1173	0.9059	0.0124
*CF231*	1.37	1.09	1.05	1.11	0.09	1.07	1.23	1.17	0.09	0.84 ^b^	1.17 ^a^	1.45 ^a^	0.09	0.0988	0.3736	0.0003
*Clostridium*	2.01	2.39	2.14	2.14	0.19	2.57 ^a^	2.08 ^ab^	1.86 ^b^	0.20	2.18	2.24	2.08	0.19	0.5747	0.0248	0.7977
*Fibrobacter*	1.17	1.22	1.45	0.77	0.19	1.27	1.40	0.79	0.19	0.65 ^b^	1.03 ^b^	1.77 ^a^	0.19	0.1219	0.0500	0.0006
*Lachnospiraceae__other_*	1.74 ^ab^	2.06 ^a^	1.51 ^b^	1.71 ^ab^	0.10	1.94	1.69	1.64	0.11	1.55 ^b^	1.72 ^ab^	2.00 ^a^	0.11	0.0244	0.0971	0.0169
*Prevotella*	17.61	16.13	21.12	16.77	1.21	16.05	18.12	19.54	1.04	17.90	16.80	19.00	1.04	0.0500	0.0893	0.3279
*Ruminococcus*	2.01	2.69	2.08	1.67	0.25	2.14	2.12	2.07	0.22	1.89 ^ab^	1.73 ^b^	2.70 ^a^	0.22	0.0703	0.9772	0.0136
*Succiniclasticum*	3.12	4.21	3.29	2.9	0.38	3.66	3.39	3.09	0.33	1.39 ^b^	4.20 ^a^	4.55 ^a^	0.33	0.1217	0.4897	<0.0001
*Treponema*	1.14	1.25	1.22	1.14	0.16	1.69 ^a^	1.08 ^b^	0.80 ^b^	0.14	0.93	1.25	1.38	0.14	0.9407	0.0011	0.0941
*Uncl_BS11*	2.40 ^a^	1.16 ^b^	2.01 ^ab^	2.62 ^a^	0.30	1.76	2.45	1.94	0.25	2.36	2.13	1.66	0.25	0.0121	0.1627	0.1610
*Uncl_Lachnospiraceae*	7.92 ^a^	8.16 ^a^	6.10 ^ab^	5.51 ^b^	0.52	7.27	6.72	6.78	0.45	6.18	7.68	6.91	0.45	0.0047	0.6510	0.0919
*Uncl_Paraprevotellaceae*	1.52 ^ab^	1.25 ^a^	1.46 ^a^	0.82 ^b^	0.10	1.15	1.32	1.31	0.08	1.05	1.28	1.46	0.08	0.0011	0.9407	0.0941
*Uncl_RFP12*	0.74	0.68	0.52	0.63	0.10	0.54	0.82	0.57	0.09	0.45 ^b^	0.69 ^ab^	0.80 ^a^	0.09	0.4777	0.0620	0.0318
*Uncl_Ruminococcaceae*	5.54	5.52	6.01	7.72	0.75	5.47	7.05	6.07	0.65	7.66 ^a^	6.09 ^ab^	4.84 ^b^	0.65	0.1697	0.2577	0.0247
*Uncl_Veillonellaceae*	0.38 ^b^	0.58 ^ab^	1.21 ^a^	0.71 ^ab^	0.18	0.65	0.75	0.76	0.15	0.53	0.87	0.77	0.15	0.0246	0.8518	0.2946
*YRC22*	1.25 ^b^	1.20 ^b^	2.09 ^a^	1.47 ^b^	0.09	1.40	1.54	1.56	0.08	1.11 ^b^	1.61 ^a^	1.80 ^a^	0.08	<0.0001	0.3094	<0.0001

^1^ Uncl: unclassified. Means with different superscript letters (a, b, c) in each row indicate significant differences (*p* ≤ 0.05) between donor cows, daily time intervals, and sampling weeks.

**Table 5:** Spearman’s correlations between the relative abundance of rumen microbiota floating in the liquid fraction and the concentration of volatile fatty acids, enzymes activities, total tract neutral detergent fiber digestibility (ttNDFDe), and total tract dry matter digestibility (ttaDMDe). The green cells indicate significant positive correlations, and the red cells indicate significant negative correlations.

	Amylase	Cellulase	Xylanase	Lactic	Acetic	Propionic	Butyrric	ttNDFDe	ttaDMDe
**Phylum**									
Actinobacteria	-	-	-	-	-	-	-	-	0.42
Bacteroidetes	-	-	-	0.39	-	-	-	-	0.35
Cyanobacteria	-	-	-	0.37	-	-	-	-	−0.41
Euryarchaeota	-	-	-	-	-	-	-	-	-
Fibrobacteres	0.56	-	-	-	-	-	-	-	-
Firmicutes	−0.45	-	-	0.43	-	-	-	-	0.34
Lentisphaerae	-	-	-	-	-	-	-	−0.48	−0.52
Proteobacteria	-	-	-	-	-	-	-	-	−0.41
Spirochaetes	0.36	-	-	-	-	-	-	-	−0.48
SR1	-	-	-	-	-	-	-	-	-
Tenericutes	0.41	-	-	-	-	-	-	−0.34	−0.40
TM7	-	-	-	-	-	-	-	−0.45	−0.44
Verrucomicrobia	-	-	-	-	-	-	-	-	-
**Family**									
*Anaeroplasmataceae*	0.44	-	-	-	-	-	-	-	-
*Bacteroidales other*	-	-	-	-	-	-	-	0.38	0.42
*BS11*	-	-	0.36	-	−0.38	−0.35	-	-	-
*Clostridiaceae*	-	-	−0.39	−0.53	-	-	−0.38	-	-
*Coriobacteriaceae*	-	-	0.36	-	-	-	-	-	0.39
*Erysipelotrichaceae*	-	-	-	-	-	-	-	-	-
*F16*	-	-	-	-	-	-	-	−0.46	−0.44
*Fibrobacteraceae*	0.56	-	-	-	-	-	-	-	-
*Lachnospiraceae*	-	-	−0.49	−0.53	-	-	−0.49	-	-
*Moraxellaceae*	0.44	-	-	-	-	-	-	-	-
*Planococcaceae*	-	-	-	-	0.35	-	-	-	-
*Prevotellaceae*	-	-	-	-	-	-	-	-	-
*RF16*	0.38	-	-	-	-	0.38	0.39	-	-
*RFP12*	0.36	-	-	-	-	-	-	-	-
*Ruminococcaceae*	−0.46	-	-	-	-	-	-	0.43	0.48
*Spirochaetaceae*	0.42	-	-	-	-	-	-	-	−0.51
*Uncl_Bacteroidales* ^1^	−0.38	-	-	-	-	-	-	-	-
*Uncl_Clostridiales*	-	-	-	-	-	-	-	0.44	0.47
*Uncl_SR1*	-	-	-	-	-	-	-	-	-
*Uncl_YS2*	-	-	-	0.36	-	-	-	-	−0.41
*Veillonellaceae*	-	0.39	-	-	-	-	-	−0.39	-
*Victivallaceae*	-	-	-	-	-	-	-	−0.47	−0.54
**Genus**									
*Acinetobacter*	0.45	-	-	-	-	-	-	-	-
*Anaeroplasma*	0.45	-	-	-	-	-	-	-	-
*Butyrivibrio*	-	-	-	−0.50	-	-	−0.37	-	-
*CF231*	0.35	-	-	0.36	-	-	-	-	-
*Clostridium*	-	-	-	−0.52	-	-	−0.36	-	-
*Coprococcus*	-	-	-	−0.41	-	-	-	-	-
*Fibrobacter*	0.56	-	-	-	-	-	-	-	-
*Lachnospiraceae other*	-	-	-	-	-	-	−0.50	-	-
*Prevotella*	-	-	-	-	-	-	-	-	-
*Ruminococcus*	-	-	-	-	-	-	-	-	-
*Succiniclasticum*	-	0.34	-	-	-	-	-	-	-
*Treponema*	0.42	-	-	-	-	-	-	-	−0.51
*Uncl_BS11*	-	-	0.36	-	−0.38	−0.38	-	-	-
*Uncl_Lachnospiraceae*	-	-	−0.48	−0.52	-	-	−0.42	-	-
*Uncl_Paraprevotellaceae*	-	-	-	-	-	0.35	-	0.43	0.43
*Uncl_RFP12*	0.36	-	-	-	-	-	-	-	-
*Uncl_Ruminococcaceae*	−0.46	-	-	-	-	-	-	0.41	0.48
*Uncl_Veillonellaceae*	-	-	-	-	-	-	-	−0.38	-
*Uncl_Victivallaceae*	-	-	-	-	-	-	-	−0.47	−0.53
*Uncl_Prevotellaceae*	−0.41	-	-	-	-	-	-	-	−0.51
*Uncl_F16*	-	-	-	-	-	-	-	−0.47	−0.44
*Uncl_RF16*	0.37	-	-	-	-	0.38	0.39	-	-
*YRC22*	-	-	-	−0.40	-	-	-	-	-

^1^ Uncl: unclassified.

**Table 6:** Spearman’s correlations between the relative abundance of rumen microbiota adhered to the solid fraction and the concentration of volatile fatty acids, enzymes activities, total tract neutral detergent fiber digestibility (ttNDFDe), and total tract dry matter digestibility (ttaDMDe). The green cells indicate significant positive correlations, while the red ones indicate significant negative correlations.

	Amylase	Cellulase	Xylanase	Lactic	Acetic	Propionic	Butyrric	ttNDFDe	ttaDMDe
**Phylum**									
Actinobacteria	-	-	0.47	-	-	-	-	-	-
Bacteroidetes	-	-	0.54	-	-	-	-	-	-
Cyanobacteria	-	-	-	-	-	-	-	0.56	0.50
Euryarchaeota	−0.41	-	-	-	-	-	-	-	-
Fibrobacteres	-	-	-	-	-	-	-	-	-
Firmicutes	-	-	−0.46	-	-	-	-	0.37	-
Lentisphaerae	-	-	-	-	-	-	-	-	-
Other	-	-	-	-	-	-	-	-	-
Proteobacteria	-	-	-	-	-	-	-	-	-
Spirochaetes	-	-	-	−0.46	-	-	-	-	-
SR1	−0.38	-	-	-	-	-	-	-	-
Tenericutes	-	-	-	-	-	-	-	-	-
TM7	-	-	-	-	-	-	-	-	-
Verrucomicrobia	-	-	-	-	-	-	-	0.37	-
**Family**									
*Anaeroplasmataceae*	-	-	-	−0.36	-	-	-	-	-
*Bacteroidales other*	-	-	0.38	-	-	-	-	-	0.47
*BS11*	-	-	0.33	-	-	-	-	-	-
*Clostridiaceae*	-	-	-	−0.35	-	-	-	-	-
*Clostridiales other*	-	-	−0.35	-	-	-	-	-	-
*Coriobacteriaceae*	-	-	0.40	-	-	-	-	-	0.36
*Erysipelotrichaceae*	-	-	-	0.40	-	-	-	-	-
*F16*	-	-	-	-	-	-	-	-	-
*Fibrobacteraceae*	-	-	-	-	-	-	-	-	-
*Lachnospiraceae*	-	-	-	-	0.34	-	-	0.38	-
*Moraxellaceae*	0.39	-	-	-	-	-	-	-	-
*Paraprevotellaceae*	-	-	0.47	-	-	-	-	-	0.37
*Planococcaceae*	-	-	-	-	-	-	-	−0.46	−0.38
*Prevotellaceae*	-	-	0.57	-	-	-	-	-	-
*RF16*	-	-	-	-	-	-	-	-	-
*RFP12*	-	-	-	-	-	-	-	0.43	-
*Ruminococcaceae*	-	-	-	-	-	-	-	-	-
*Spirochaetaceae*	-	-	-	−0.48	-	-	-	-	-
*Uncl_Bacteroidales* ^1^	-	-	-	-	-	-	-	0.43	0.45
*Uncl_Clostridiales*	-	-	-	-	-	-	-	-	-
*Uncl_SR1*	−0.38	-	-	-	-	-	-	-	-
*Uncl_YS2*	-	-	-	-	-	-	-	0.56	0.49
*Veillonellaceae*	-	-	-	-	-	-	-	0.43	0.49
Victivallaceae	-	-	-	-	-	-	-	-	-
**Genus**									
*Acinetobacter*	0.36	-	-	-	-	-	-	-	-
*Anaeroplasma*	-	-	-	−0.40	-	-	-	-	-
*Butyrivibrio*	-	-	-	-	-	-	-	-	0.39
*CF231*	−0.47	-	-	-	-	-	-	-	0.38
*Clostridium*	-	-	-	−0.35	-	-	-	-	-
*Coprococcus*	-	-	-	-	-	-	-	-	-
*Fibrobacter*	-	-	-	-	-	-	-	-	-
*Lachnospiraceae other*	-	-	−0.37	-	-	-	-	0.34	0.36
*Prevotella*	-	-	0.58	-	-	-	-	-	-
*Ruminococcus*	-	-	-	-	-	-	-	-	-
*Succiniclasticum*	-	-	-	-	-	-	-	0.47	0.49
*Treponema*	-	-	-	−0.49	-	-	-	-	-
*Uncl_BS11*	-	-	0.33	-	-	-	-	-	-
*Uncl_Lachnospiraceae*	-	-	-	-	0.42	0.34	-	0.38	-
*Uncl_Paraprevotellaceae*	-	-	-	-	-	-	-	-	-
*Uncl_RFP12*	-	-	-	-	-	-	-	0.43	-
*Uncl_Ruminococcaceae*	-	-	-	-	-	-	-	-	-
*Uncl_Veillonellaceae*	-	-	0.40	-	-	-	-	-	0.36
*Uncl_Victivallaceae*	-	-	-	-	-	-	-	-	-
*Uncl_Prevotellaceae*	-	-	-	-	−0.37	-	−0.38	-	0.34
*Uncl_F16*	-	-	-	-	-	-	-	-	-
*Uncl_ RF16*	-	-	-	-	-	-	-	-	-
*YRC22*	-	-	0.46	-	-	-	-	0.34	0.45

^1^ Uncl: unclassified.

## Data Availability

All data and analyses in this study are included in this published article and its supplementary files.

## Supplementary Materials

The following supporting information can be downloaded at: https://www.mdpi.com/article/10.3390/ani14233547/s1, Figure S1: Barplots of taxa relative abundances at phylum, family and genus level for the liquid fraction of the rumen; Figure S2: Barplots of taxa relative abundances at phylum, family and genus level for the solid fraction of the rumen. Table S1. Total tract neutral detergent fiber digestibility, apparent dry matter digestibility (% DM), concentrations of acetic, propionic, butyric and lactic acid (mg/100 mL), and enzymatic activities of amylase, cellulase, and xylanase (expressed as area of hydrolysis of the rumen fluids—RFs) in rumen fluid of the four donor cows (DC) at 0 h (before feeding), 4 h and 8 h after feeding, in three consecutive sampling weeks and their interactions.
