# Supplementary material for: The Effects of Animal, Collection Time, and Interval on the Microbiota Structure, Metabolism, and Degradative Potential of Rumen Fluid Inoculum Collected by Esophageal Probe from Hay-Fed Cows
Source: Animals (Basel). 2024 Dec 9;14(23):3547. doi: 10.3390/ani14233547 (PMC11639906; doi:10.3390/ani14233547)

Table S1. Total tract neutral detergent fiber digestibility, apparent dry matter digestibility (% DM), concentrations of acetic, propionic, butyric and lactic acid (mg/100 mL), and enzymatic activities of amylase, cellulase, and xylanase (expressed as area of hydrolysis of the rumen fluids - RFs) in rumen fluid of the four donor cows (DC) at 0h (before feeding), 4h and 8h after feeding, in three consecutive sampling weeks and their interactions.

	**Cow * Time**				**Cow * Week**				**P-values**	
	**Time 0**	**Time 4**	**Time 8**	**SEM**	**Week 1**	**Week 2**	**Week 3**	**SEM**	**C*T**	**C*W**
Total tract neutral detergent fiber digestibility (% DM)										
DC1	43.22ab	45.39a	43.46a	1.31	37.06f	51.35a	43.66cd	1.31	0.0054	0.0030
DC2	43.92a	46.17a	44.85a	1.31	39.16ef	49.89a	45.89bc	1.31		
DC3	46.93a	38.75c	39.38bc	1.31	40.34def	41.51de	43.21cd	1.31		
DC4	44.35a	45.91a	47.11a	1.31	40.53def	47.91ab	48.83ab	1.31		
Total tract apparent dry matter digestibility (% DM)										
DC1	72.54b	75.07a	74.17ab	0.80	71.93c	74.97ab	74.89ab	0.80	0.3515	0.8851
DC2	74.13ab	75.54a	75.53a	0.80	73.97bc	75.55ab	75.68ab	0.80		
DC3	74.44ab	73.65ab	75.42a	0.80	73.57bc	74.52ab	75.42ab	0.80		
DC4	75.53a	74.67ab	75.81a	0.80	73.64bc	75.91ab	76.45a	0.80		
Acetic acid										
DC1	479.19	405.308	395.45	58.14	401.61	486.96	391.45	58.14	0.3559	0.8174
DC2	385.94	479.91	393.4	58.14	380.35	422.67	456.24	58.14		
DC3	451.83	376.12	388.48	58.14	389.98	394.74	431.71	58.14		
DC4	338.03	273.89	435.4	58.14	368.57	370.56	308.20	58.14		
Propionic acid										
DC1	90.68	92.62	93.70	14.71	85.64	99.22	92.14	14.71	0.2381	0.9112
DC2	81.92	100.59	99.08	14.71	84.35	98.85	98.39	14.71		
DC3	108.09	75.57	100.46	14.71	85.51	92.60	106.00	14.71		
DC4	71.82	54.55	118.20	14.71	89.53	78.70	76.34	14.71		
Butyric acid										
DC1	60.14	57.7	71.17	7.99	60.05	67.46	61.49	7.99	0.5046	0.9926
DC2	39.67	62.57	63.28	7.99	51.77	57.46	56.29	7.99		
DC3	52.16	53.08	74.44	7.99	61.19	64.01	54.47	7.99		
DC4	41.79	34.73	63.47	7.99	47.59	46.82	45.58	7.99		
Lactic acid										
DC1	9.34bcde	15.62b	10.97bcd	2.23	17.30	8.85	9.78	2.23	0.0006	0.1546
DC2	3.76e	7.39cde	13.24bc	2.23	5.27	11.24	7.87	2.23		
DC3	2.28e	6.56cde	7.34cde	2.23	7.1	6.12	2.90	2.23		
DC4	2.70e	4.81de	32.40a	2.23	14.41	14.57	10.93	2.23		
Amylase										
DC1	106.76	101.26	140.16	11.07	94.40ef	164.62ab	89.15f	11.07	0.1881	0.0126
DC2	137.56	136.00	146.52	11.07	140.74bc	175.25a	104.09def	11.07		
DC3	122.79	140.43	143.96	11.07	150.05abc	132.23bcd	124.90cde	11.07		
DC4	149.22	130.61	124.05	11.07	136.86bcd	141.74bc	125.27cde	11.07		
Cellulase										
DC1	249.77	288.96	221.03	30.54	269.12	215.29	275.35	30.54	0.1175	0.3043

DC2	250.60	182.49	225.33	30.54	253.58	141.30	263.54	30.54		
DC3	232.51	292.53	191.79	30.54	260.70	226.71	229.42	30.54		
DC4	230.77	158.64	176.41	30.54	228.90	184.18	152.75	30.54		
Xylanase										
DC1	115.79	142.97	138.92	14.26	127.65	126.65	143.37	14.26	0.1538	0.4734
DC2	85.76	110.25	109.49	14.26	67.61	125.77	112.11	14.26		
DC3	144.24	197.10	187.29	14.26	176.23	193.95	132.97	14.26		
DC4	138.02	109.09	166.45	14.26	132.97	137.02	143.58	14.26		

Figure S1. Changes in the liquid rumen bacteriome relative abundance (% of the identified OTUs) between the four donor cows (DC), primiparous and multiparous donor cows, the three consecutive sampling weeks, and three sampling intervals within the day based on most abundant phyla, families, and genera.

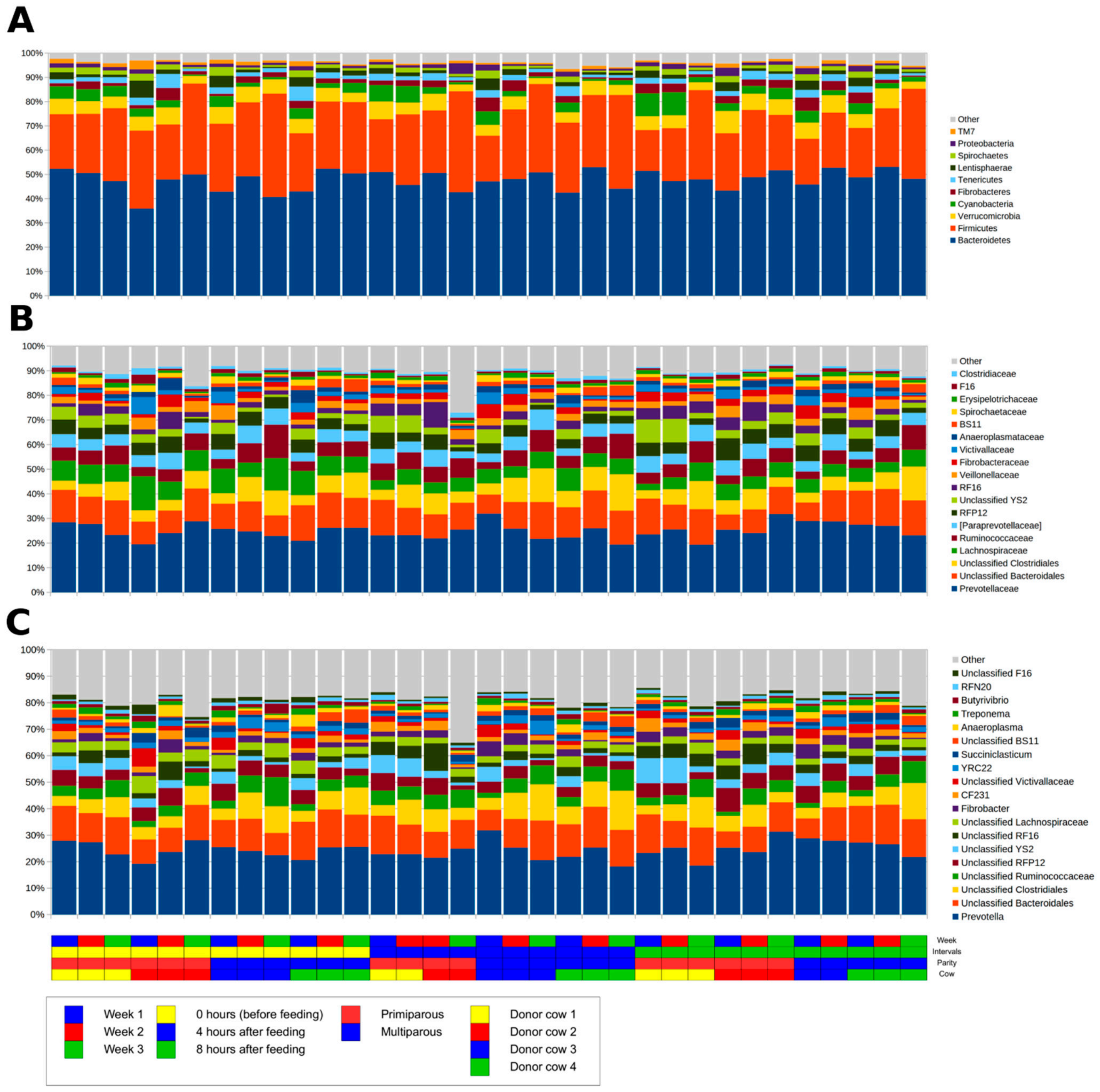

Figure S2. Changes in the solid rumen bacteriome relative abundance (% of the identified OTUs) between the four donor cows (DC), primiparous and multiparous donor cows, the three consecutive sampling weeks, and three sampling intervals within the day based on most abundant phyla, families, and genera.

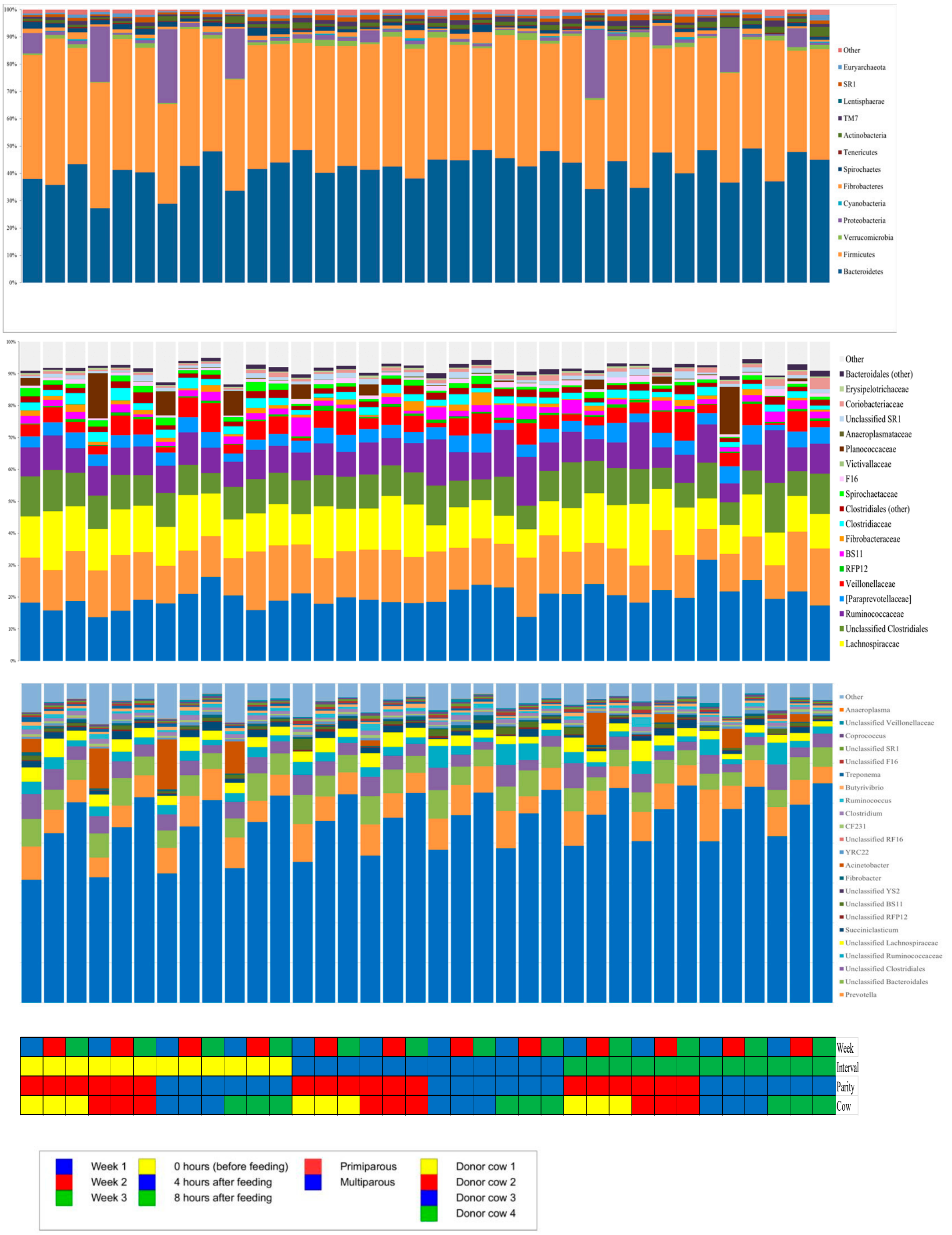

Supplement: Supplementary file 1 [file animals-14-03547-s001.zip › animals-3306619-supplementary.pdf]
